# Oncological and functional results of the surgical treatment of vertebral metastases in patients with multiple myeloma”

**DOI:** 10.1186/s12893-017-0288-9

**Published:** 2017-08-23

**Authors:** Grzegorz Guzik

**Affiliations:** 1 Orthopedic Oncology Department of the Podkarpacki Oncology Hospital, Bielawskiego 18, 36-200 Brzozów, Poland; 2 Dworska 77a, 38-420 Korczyna, Poland

**Keywords:** Metastases, Spinal tumours, Spine surgery, Resections, Stabilization, Multiple myeloma

## Abstract

**Background:**

In nearly 30% of patients with myeloma, pathological fractures are found to occur in the spine. If the patients are not treated promptly and satisfactorily, the quality of their lives diminishes. Currently, the standard treatment for metastatic lesions of the spine is radiotherapy, but surgical intervention is becoming more frequent. It is very important to quickly identify metastases and implement surgical treatment before any fracture/s occur.

**Methods:**

Over the period of 2010–2014 in our department, a total of 129 patients were treated for metastatic spinal myeloma. 73 patients underwent vertebroplasty and 56 patients were operated on through various methods. Indications for the surgery, its course, technique and outcome were subsequently evaluated. The majority of patients (76%) admitted for treatment, exhibited vertebral fractures. Most lesions were multiplace and involved the vertebral bodies. In 42% of the patients, radiological examinations showed symptoms of compression of the nervous structures, while clinical signs were observed in only 16% of the patients. The functional status of the patients was assessed using the Karnofsky scale, while pain intensity was measured in a VAS score, before and after the surgery. The oncological results were assessed as a survival rate and local recurrence rate.

**Results:**

The average follow-up was conducted within 31 months (min 18, max 48). The patients after vertebroplasty survived 42 months, and the patients after surgery 23 months. Local recurrence of the disease was observed in 12 patients. In 10 patients, among a group of 21 with paresis, their neurological conditions improved. The average results of both their VAS score and Karnofsky performance score in patients after surgery was seen to have improved. Only sporadic postoperative complications after vertebroplasty and surgery were reported.

**Conclusions:**

Early diagnosis of myeloma spine metastasis is essential to achieve the desired results of treatment. Vertebroplasty, as advised, should be performed as early as possible. Both the functional and oncological results after vertebroplasty are beneficial and the complication rates are low. Three relevant factors were found in our study: patient’s age over 65 years, initial diagnosis over 3 years and stage III of disease were related, significantly and statistically to survival.

## Background

Effective oncological treatment significantly prolonged the life expectancy of patients with multiple myeloma (MM). It is estimated that approximately 70% of sufferers have osteolytic lesions in the spine, and in 30% of these pathological fractures occur. Spinal cord compression is reported to develop in 11–24% of patients with MM [1–3].

The quality of life in patients suffering from spinal fractures is significantly decreased. Patients experience acute pain and their mobility is significantly reduced. They oftens use walking frames or crutches. Among patients with vertebral column involvement, three type of pain can be distinguished, according to such characteristical features such as: localization, radiation and exacerbating factors. Mechanical pain is associated with an instability of the vertebral column. It is a localized, sharp pain, exacerbated in the standing position, and frequently alleviated after stabilization in an orthopedic corset. Biological pain is unremitting, worse in the supine position, less posture-dependent, unresponsive to medication and more noticeable at night. Damage to the body neurons results in neuropathic pain, which is often described as shooting, traveling along the nerves. Spinal axis disorder creates the risk of adjacent vertebrae syndrom. In some patients, numerous neurological disorders may coexist. [4–6]

The treatment of multiple myeloma is a combination of chemotherapy and radiotherapy. The ultimate goal of the treatment is to achieve remission or stability of the disease. Radiotherapy of the bone and the metastases causes a decrease in the number of local recurrence and pain. However, radiotherapy alone cannot restore bone loss, strengthen their structure or increase their mechanical strength. Also, restoration of the proper shape and function of the spine, and protection from further fractures is impossible to achieve with radiotherapy alone. In the case of compression of the nervous structures, by the tumor masses, radiotherapy may result in a reduction of neurological deficits. However, effectiveness in cases where compression is caused by bone fragments is limited [2, 7, 8].

Surgical treatment of lesions caused by multiple myeloma in the spine is controversial and has both advantages and disadvantages. The mechanical stabilization of the spine is not fully-effective due to insufficient bone tissue quality, and a lack of healing reactions. Bone union after the surgical procedure of stabilization is impossible. Surgery should be reserved for cases where urgent decompression of neurological structures is imperative, and for patients in long-term remission [2, 7, 9].

Remarkably effective in treating myeloma lesions of the spine are vertebro- and kyphoplasty. These methods allow for the strengthening of the damaged vertebra, and prevent spinal kyphotisation and its further consequences. Kyphoplasty allows for the partial re-establishment of the original height of the collapsed vertebra, and therefore is effective in patients with fractures. Both methods successfully decrease pain. The treatment does not necessitate interruption in chemotherapy and procedures can be performed under local anesthesia. 3–4 vertebrae can be operated on simultaneously. Contraindications to vertebroplasty are symptoms of spinal canal stenosis, neurological deficits and haemorrhagic difficulties [2, 10–13].

The study evaluates the oncological and functional outcomes after surgical treatments of the patients with multiple myeloma involving the vertebral column.

## Methods

Over the period 2010–2014, a total of 542 patients with spinal tumours were treated in our facility, of which 474 were operated on. In 129 patients the indication for surgery was multiple myeloma. The majority of patients 82/129 (64%) were women. The average age of the women was 72, and 68 years for the men.

**Fig. 1:**
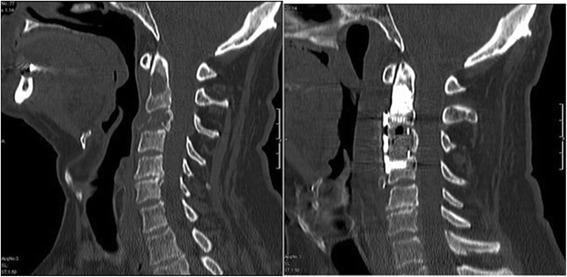
Myeloma metastasis to C2 and C3 (**a**) and radiograms after surgery (vertebral body prosthesis of C3 and the plate fixation, dens axis vertebroplasty) (**b**)

The most common site of involvement by widespread metastatic lesions was the thoracic spine. Metastases in the lumbar spine were less common. In the cervical spine, there was only one patient with a tumor in the second and the third body of the vertebra – Fig. 1.

Isolated lesions involving single vertebrae were rare; remarkable only in 10 out of 129 (8%) patients. The regions most often invaded by metastases were the vertebral bodies (98/129 patients), less frequently the posterior elements. Soft tissue masses were noted in 31 out of 129 (24%) patients. Spinal canal stenosis occurred in 54/129 (42%) patients. Pathological fractures were diagnosed in 98 of the 129 (76%) patients. Symptoms of spinal instability were seen to afflict 80/129 (62%) of the patients.

The functional results of patients were evaluated according to the Karnofsky scale, and pain intensity was measured in a VAS score before and after the surgery.

**Fig. 2:**
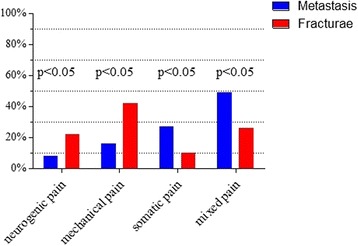
The type of pain in patients depending on a pathological fracture of the vertebral column

The pain suffered by the patients was of various characters and degrees, it was caused by the instability of the spinal column as a component factor in 75 out of 129 (58%) patients, and in 39 out of 129 patients (30%), the causes were neuralgic. Biological pain brought on by by increased intraosseous pressure, occurred in 48 out of 129 (37%) patients. The severity of this pain was assessed in the patients prior to surgery and on postoperative day 7 and 14, using VAS. More severe symptoms were observed in the patients with vertebral fractures and spinal instability – Fig.2.

The study also assessed the pre- and post-operative neurological status of patients with special consideration being taken concerning the strength of the muscles, sensory abilities, sphincters function, and the occurrence of symptoms from compressed nerve roots. Table 1 demonstrates baseline clinical characteristics and examination findings in patients with myeloma metastases of the vertebral column.

**Table 1:** Baseline clinical characteristics and examination findings in patients with myeloma metastases in the vertebral column. *N* = 129

Variables	*N* (%)
Fracture	98 (76)
Multi level involvement	119 (92)
Vertebral body involvement	98 (76)
Spinal canal stenosis	54 (42)
Soft tissue mass	31 (24)
Orthopedic examination elements	
Spinal axis disorder	68 (53)
Reflex scoliosis	61 (47)
Increased paraspinal muscle tension	94 (73)
Spinal pain on spinous processes	112 (87)
Pain on axial compression by pressing the head	86 (67)
Reduced mobility of the spine	114 (88)
Pain on movement	98 (76)

Results are presented as a number with a percentage

A total of 129 patients with myeloma metastases of the vertebral column underwent surgery.

A spine fixation was performed in 56 patients. Four patients underwent resection of the vertebral body, in conjunction with prosthesis implantation and lateral stabilization. In 8 patients, the surgery was performed through 2 approaches, and involved vertebral body resection, prosthesis implantation and percutaneous stabilization of the spine through the posterior approach. Forty four patients were operated on through the posterior approach. Extensive laminectomy, combined with stabilization of the spine was performed. Each time, stabilization encompassed two vertebrae above and two vertebra below the malignant lesion. Indications for open spinal surgery were: the presence of a large soft tissue mass, neurological deficits, spinal instability, central or lateral stenosis, extensive spinal axis disturbances and vertebral collapse over 50%. Generally, open spinal surgery was performed when vertebroplasty was contraindicated, and patients were in good general condition. Contraindications to the open surgical proceedure were: a patients poor overall health condition, a life expectancy of less than 3 months, active infection at the site of surgical access.

Vertebroplasty was performed in 73 patients. In total, 112 vertebrae were cemented - maximum of 3 vertebrae at the same time. The procedures were performed under general anaesthesia in 63 out of 73 (86%) patients and under local anaesthesia in 10/73 (14%) patients. The needle was always inserted one-sidedly into the vertebral body, through the pedicle and under X-ray guidance. Cementation through the bilateral approach was not performed. The patients scheduled for vertebroplasty were those with metastases with no fractures, or with fractures but without any substantial deformations of the spine. It was assessed whether there were no losses in the anterior wall of the spinal canal. Patients with vertebral compression (over 50% of the initial group) and patients with recently experienced neurological symptoms or spinal canal stenosis were not scheduled for a vertebroplasty.

Adjuvant radiation therapy was administered to 47 out of 73 (64%) patients after vertebroplasty and 38/56 (68%) patients after surgical intervention.

Quantitative variables were expressed as means (x) with standard deviations. To compare the effects of different treatments, an options paired Student’s t-test was used. The sub-group differences were tested using a Wilcoxon test. The categorical variables were expressed as percentages. The inter-group differences were tested using the χ2 test. The inter-group overall survival rates were compared with a Log-Rank test and presented as the Kaplan–Meier curves. All statistical analyses were performed by employing Statistica 10. A value of *P* < 0.05 was considered statistically significant.

The research was performed in accordance with the declaration of Helsinki. As this retrospective analysis consists of anonymised clinical routine data, the Research Ethics Committee deems the application for and issue of Ethics approval, as not necessary. All the patients gave their written consent to the use of data for our research.

Contact information of Ethics committee: Ethics Committee in Cracov, ul Krupnicza 11a 31–123 Cracov, tel. +48,126,191,712, fax +48,124,225,755.

## Results

**Fig. 3:**
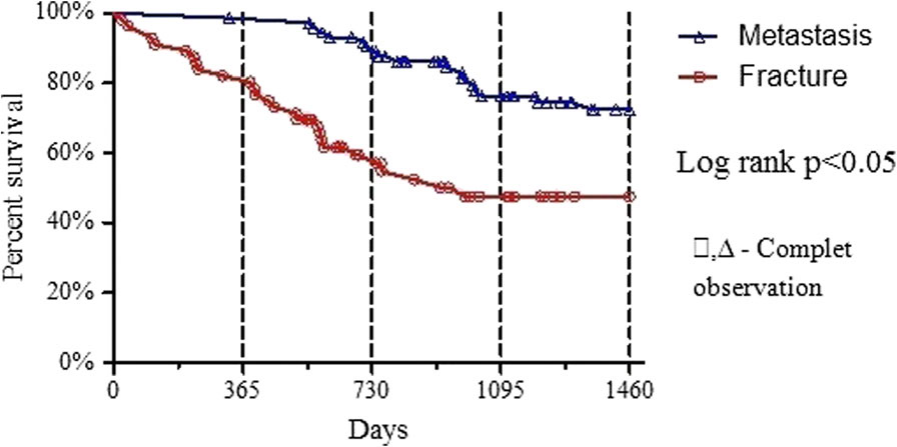
Survival of the patients according to type of vertebral involvment. *N* = 129

The average follow-up was 31 months (min 18, max 48). In the final review it was found that 53 patients after vertebroplasty had survived, compared to 27 after surgery. The median survival was 34 months. On average, patients after vertebroplasty survive 42 months compared to 23 after surgery. Survival rates are exhibited in Fig. 3, according to the type of vertebral involvement. Local recurrence of the disease was observed in 12 patients who underwent surgery.

It was assessed whether the following factors affect survival: age, time of initial diagnosis, post-operative radiotherapy, stage of disease and biochemical markers. Three significant factors (age over 65 years, initial diagnosis over 3 years and III stage of disease) were statistically related to survival – Table 2.

**Table 2:** Analysis of numerous factors that might be related to overall survival

	Number of patients	*p*-value
Age		
< 65 years	43 (33%)	<0.05
> 65 years	86 (67%)	<0.05
Stage of disease (Salmon-Durie)		
I	8 (6%)	Ns
II	20 (16%)	Ns
III	101(78%)	<0.05
Radiotherapy		
Yes	85 (66%)	Ns
No	44 (34%)	Ns
Time to initial diagnosis		
< 1 year	23 (8%)	Ns
1–2 years	48 (37%)	Ns
> 3 years	71 (55%)	<0.05
CRP level		
< 5 mg/l	91 (70%)	Ns
> 5 mg/l	38 (30%)	Ns
Peripheral blood stem cell transplantation		
Yes	71 (55%)	Ns
No	58 (45%)	Ns
Lactate dehydrogenase		
< 240 U/l	93 (72%)	Ns
> 240 U/l	36 (28%)	Ns
Beta-2 microglobulin		
< 3.5 mg/l	8 (6%)	Ns
3.5–5.5 mg/l	31 (24%)	Ns
> 5.5 mg/l	90 (70%)	Ns

The mean duration of the surgeries, utilizing the anterior approach, was 120 min (range: 90–170 min), and 80 min (range: 50–120 min) employing the posterior approach. The average duration of the surgeries through both of the approaches was 220 min (range: 120–300 min). Patients were hospitalized between 8 and 22 days (14 days on average). The mean vertebroplasty time was 35 min (range: 25–60). The hospital stay time was 2 days, on average. There was also an assessment of the frequency of the occurrence of potential contraindications to vertebroplasty – Table 3.

**Table 3:** Frequency of potential contraindications to vertebroplasty in our group of patients

Potential contraindications to vertebroplasty	Patients qualified to vertebroplasty *n* = 73 *n*/%	Patients qualified to spine stabilizations *n* = 56 *n*/%
Impairment of the neural elements	No	21(37%)
Cauda equine syndrome	No	6(11%)
Spine kyphosis	12(16%)	56 (100%)
Fractures with obstructing plasmocytoma	No	31(55%)
Retropulsed bone	No	56(100%)
Vertebra plana	No	15(27%)
Disruption of the posterior vertebral body cortex	No	54(96%)
Unstable spine	47 (64%)	56(100%)
Metal or PMMA alergy	No	No
Coagulopathy	No	No
Neutropenia	6(8%)	2(4%)
Cardio-pulmonary insufficiency	No	No
Radiotherapy before surgery	2(3%)	4(7%)
Skin infections	No	No

The functional results were evaluated as, the pain rate in a VAS score and performance in a Karnofsky score – Table 4.

**Table 4:** Mean results of the VAS score and the Karnofsky performance score in patients before and after surgery, and after different treatment methods

Treatment option	VAS score		Karnofsky score	
	Preoperative	Postoperative	Preoperative	Postoperative
Patients after vertebroplasty *N* = 73	5,3 (±1.8)	1,4 (±0.9)*	60 (±7.9)	80 (±5.6)*
Patients after stabilization *N* = 56	8.1 (±0.6)	4.4 (±1.1)*	54 (±9.8)	59 (±12.2)
Anterior stabilization *N* = 4	7.5 (±1.2)	4.7 (±1.0)	52 (±8.0)	58 (±9.2)
Posterior stabilization *N* = 44	8.0 (±0.4)	4.1 (±0.6)*	56 (±5.5)	62 (±5.5)
Combine stabilization *N* = 8	9.2 (±1.6)	4.5 (±0.9)*	49 (±11)	55 (±10)
Vertebroplasty vs. Stabilization	p = <0.05	p = <0.05	NS	*p* = <0.05

Results are presented as a mean ± standard deviation

* *p* < 0.05

In 10 patients out of 21 with paresis, neurological functions improved. Neurological status were described in Table 5.

**Table 5:** Neurological status in patients before and after the surgery, and after different treatment methods

Neurological Status	Posterior stabilization *N* = 44		Combine stabilization *N* = 8	
	Preoperative	Postoperative	Preoperative	Postoperative
Frankel A	5 (11)	3 (7)	1 (12)	0
Frankel B	5 (11)	2 (5)*	0	1 (12)
Frankel C	3 (7)	2 (5)	1 (12)	1 (12)
Frankel D	2 (5)	0	0	0
Sensory impairments	4 (9)	2 (5)*	0	0

Results are presented as a number with a percentage

* *p* < 0.05 χ^2^

There were infrequent postoperative complications after vertebroplasty: asymptomatic cement leakage into the spinal canal occurred in two patients and pulmonary microembolism in one patient. After the surgery, 3 patients suffered from impaired wound healing, and two required revision surgery. The wounds healed in all of the patients. There was no deterioration in neurological status after the surgery, and there was no evidence of loosening or mechanical damage to the surgical implants – Table 6.

**Table 6:** Systemic complication after surgery in patients with myeloma metastases in thevertebral column

Treatment option	Systemic complication		
	Surgical wound infections	Pulmonary embolism	Local recurrence
Patiens after vertebroplasty *N* = 73	0	1 (1.3)	0
Patients after stabilization *N* = 56	3 (5)	2 (3.6)	12 (22)

Results are presented as a number with a percentage

## Discussion

Myeloma is a haematopoietic malignancy, particularly frequently diagnosed in the 6-7th. decade of life. Diagnosis of myeloma is based on radiological examinations and on detection of monoclonal immunoglobulines or light chains in the urine. Confirmation of a diagnosis is based on the histopathological examination of the patient’s bone marrow, following sternum or iliac crest trepanobiopsy, supplemented with an evaluation of the tumour [2, 12, 13]. In the last few years many authors have worked to determine the suitability of KISS1R for myeloma multiplex diagnosis. In the future it could be usefull for detecting elary changes in the bone microenvironment and the patient’s response to treatment [14]. The treatment of myeloma consists of polychemotherapy complemented with radiotherapy. This allows for long-term remissions, even for many years. In the past, spinal lesions were treated non-surgically, with radiotherapy only [2, 12]. The efficiency of radiotherapy in palliating pain was 75–100% in several studies. Uni and multivariate binary logistic regresion analysis improved pain relief, was closely associated with the patients age and the total radiotherapy doses. Recalcification was observed in 48% of the treated cases and was associated with the total radiotherapy doses [15, 16]. Lecouvet et al. reported new focal marrow lesions in 4% of irradiated and 27% of non-irradiated vertebras and new fractures in 5% of irradiated and 20% of non-irradiated vertebras [17]. Currently, various methods of surgical treatment are increasingly being employed and radiotherapy is seen as an adjuvant. This creates the greatest opportunities for alleviating pain and adaquate physical functioning for the patient’s long-term [2, 18].

The multiple myeloma is localized particularly often in the thoracic and lumbar region of the spinal column; within its vertebral bodies. The posterior elements are rarely involved. What is characteristic, apart from lytic bone lesions, is the occurrence of soft tissue masses which compress the adjacent organs or neurologic structures [2, 12]. Among other factors, this is the cause of the combination of two or three different types of pain in some patients. We have reported the frequent occurrence of neuropathic pain in patients without vertebral fractures. Also spine instability, that causes mechanical pain, in patients without fractures, is associated with damage to the spinal ligaments and spinal muscles.

It is imperative for adequate treatment, to diagnose the lesions as early as possible, in the vertebral column. In more than 90% of patients a clinical examination allows for the diagnosis of metastases in the spine. Radiological examinations permit an accurate assessment of their location and size, as well as aiding in treatment planning. In the case of minor lesions, it is sufficient to conduct a radiographic examination and CT scan of the vertebral column. Large soft-tissue tumours, and those causing neurological deficits, require diagnosis through MRI [2, 19–21].

Qualification for treatment is multifaceted and requires the cooperation of the orthopedic surgeon, hematologist, radiologist, anesthesiologist, and radiotherapist. It is important to establish a prognosis, estimate the stage of the disease, assess the general condition of the patient, discover any chronic diseases and the possibility of complementary therapies.

Patients with lytic lesions in the vertebrae should be scheduled for vertebroplasty as early as possible, before pathological fractures can occur. This allows for the retention of the normal shape of the vertebra and the whole vertebral column, successfully eliminates pain associated with malignant disease and posture pain [2, 22, 23]. Kado et al. showed statistically significant mortality decreased in patients without vertebral fractures, compared to patients with five or more fractures [24]*.* Patients with fractures and no major spinal deformities should also be scheduled for vertebroplasty or kyphoplasty as soon as possible. After the surgery, adjuvant radiotherapy can be given to limit the progression of the disease [2, 25, 26]. In their study Dudeney et al. demonstrated that over 80% of patients experienced pain relief after vertebral augmentation [10].

Vrionis et al. in his study reported the significant difference in pain intensity after vertebroplasty in 95% of patients. Cement leakage was detected in 13% of patients [27]. Fourney et al. reported the complete pain reduction in 84% of patients after vertebroplasty. Cement leakage was detected in 9.2% of the patients [28].

In our group of patients, 78% were operated on in the stage III of the disease. Open spine surgeries and vertebroplasty in the early stages of the disease were rare. The reason may be due to a lack of clear indications for different treatment methods including radiotherapy, vertebroplasty and surgery. Oncologist, Hematologist and Orthopedists have different experiences in the treatment of myeloma. Furthermore, patients often decline to undergo preventive surgical treatment.

Patients with large tumors causing pressure on the nerve structures, the destruction of major bones and damage to the anterior wall of the vertebral canal, as well as spinal instability, are scheduled for decompression surgery and stabilization of the spine. It should be kept in mind that the risk of complications in patients who underwent surgery immediately following radio- and chemotherapy is high. The widest possible decompression of neural structures creates an opportunity to reverse the neurological deficits. Multi-segmental stabilization of the spine is recommended, as it allows for the rehabilitation of the patients immediately after surgery. Bone fusion is unlikely to occur. It is generally agreed to avoid the use of bone grafts due to the significantly increased risk of wound infection. Radical excision is possible when the tumours are well demarcated. In place of the removed vertebral body, a titanium prosthesis or bone cement may be inserted [2, 29–33]. The majority of studies for open surgery qualified patients with neurological defects - 81% of patients [34]. After surgical treatment of metastases from multiple myeloma, many studies have reported an increase in the quality of life and survival of their patients. Complete pain reduction was observed in 76–100% of patients, and neurological status improvement in 50–76% of patients [34–36]. Zeifang et al. and Rompe et al. showed neurological improvement in up to 80% of patients with myeloma, following the combined anterior and posterior approaches. 91% of patients confirmed a reduction in pain, and 57% experienced improvement in mobility [36, 37]. Total complication rates after spinal surgery were ranked from 10 to 52% [34, 38]. Pascal-Moussellard et al. reported the observance of 19% of complications after surgery, with the most common being difficulty in wound healing - 11% and infections - 10%. Trombo-embolic complications occurred in 11% of patients. Neurological function detoriation was reported in 3% of patients. No damage to implants was observed. [39].

In our patient group the incidence of complications were very low. This was probably related to our respect of the indications and contraindications to different surgical techniques. The correct and careful surgical technique, experience and good cooperation of the therapeutic team, seems to be the key to obtaining desirable treatment results. Very important is the proper visualisation of the tumour morphology in CT and MRI scans. It allows for the planning of the operating technique details, and for the avoidance of complications. In our study the best results were achieved by patients without spine fractures, who were treated by vertebroplasty. The pain intensity rated on a VAS score, Karnofsky performance status and survival were significantly increased. The reason may be due to the qualification of patients to vertebroplasty, in the earlier stages of the disease than to open spine surgery. Age over 65 years, time to initial diagnosis over 3 years and III stage of disease, were remarked as being statistically related to survival. The author postulates that the early diagnosis of spinal myeloma metastases is a condition necessary for the achievement of a successful treatment outcome.

## Conclusions

Multiple myeloma spine metastases treatement requires a multidisciplinary, comprehensive approach and early diagnosis which is essential to achieve the desired results of treatment. Vertebroplasty, as advised, should be performed as early as possible. Both the functional and oncological results after vertebroplasty are beneficial and the complication rates are low. Three relevant patient factors: age over 65 years, initial diagnosis over 3 years and III stage of disease were significantly and statistically related to survival.

## Abbreviations

BJP: Bence Jones Proteins
ESR: Erythrocyte Sedimentation Rate
PMMA: Polymethyl methacrylate
VAS: Visual Analogue Scale
